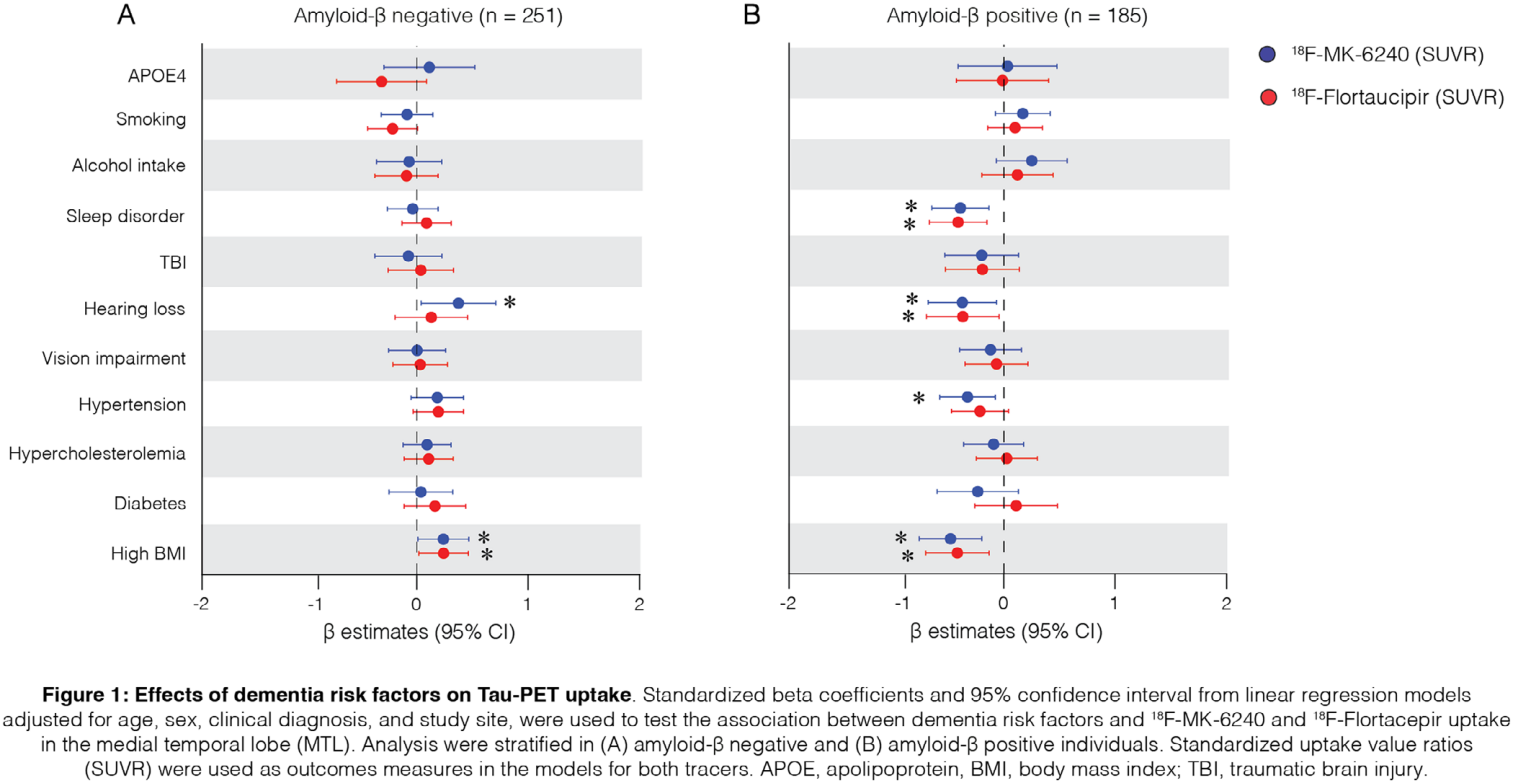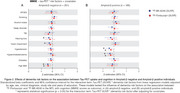# Effects of AD modifiable risk factors to tau‐PET tracer uptake and its association with cognitiion in early Braak stages

**DOI:** 10.1002/alz70856_106398

**Published:** 2026-01-07

**Authors:** Matheus Scarpatto Rodrigues, Bruna Bellaver, Guilherme Povala, Guilherme Bauer‐Negrini, Firoza Z Lussier, Livia Amaral, Pamela C.L. Ferreira, Markley Silva Oliveira, Andreia Rocha, Pampa Saha, Marina Scop Madeiros, Carolina Soares, Emma Ruppert, Rayan Mroué, Joseph C. Masdeu, Dana L Tudorascu, David N. Soleimani‐Meigooni, Juan Fortea, Val J Lowe, Hwamee Oh, Belen Pascual, Brian A. Gordon, Pedro Rosa‐Neto, Suzanne L. Baker, Tharick A Pascoal

**Affiliations:** ^1^ University of Pittsburgh, Pittsburgh, PA, USA; ^2^ Universidade Federal do Rio Grande do Sul, Porto Alegre, Brazil; ^3^ Houston Methodist Research Institute, Houston, TX, USA; ^4^ University of California, San Francisco, San Francisco, CA, USA; ^5^ Sant Pau Memory Unit, Hospital de la Santa Creu i Sant Pau, Biomedical Research Institute Sant Pau, Barcelona, Spain; ^6^ Mayo Clinic, Rochester, MN, USA; ^7^ Brown University, Providence, RI, USA; ^8^ Houston Methodist Neurological Institute, Houston, TX, USA; ^9^ Washington University in St. Louis, School of Medicine, St. Louis, MO, USA; ^10^ McGill University Research Centre for Studies in Aging, Montreal, QC, Canada; ^11^ Lawrence Berkeley National Laboratory, Berkeley, CA, USA

## Abstract

**Background:**

Many risk factors can contribute to the occurrence of Alzheimer`s Disease (AD). However, little is known about the impact of dementia risk factors to the uptake of tau‐PET tracers. Therefore, in this work we aim to investigate the influence of dementia risk factors and comorbidities on ^18^F‐Flortaucipir (FTP) and ^18^F‐MK6240 (MK) tau‐PET tracers’ uptake. Additionally, we will assess how these factors impact the association of tau‐PET and cognition.

**Method:**

We accessed 436 individuals across the aging and AD spectrum (251 amyloid negative and 185 amyloid positive) from the HEAD study, with available Aβ‐PET, FTP, MK, and clinical assessments. Linear regression models corrected for age, sex, clinical diagnosis, and study site tested the association of factors with tau‐PET tracers in the medial temporal lobe (MTL). A tau‐PET × risk factor term was added to test the influence of risk factors to the association of tau with cognition.

**Result:**

In amyloid‐β negative individuals, high BMI were positively associated with the uptake of both FTP and MK, whereas hearing loss were positively associated only with MK in the MTL (Figure 1A). In amyloid‐β positive individuals, high body mass index (BMI), hearing loss and sleep disorders were negatively associated with the uptake of both tau‐PET tracers in the MTL. On the other hand, hypertension showed negative association only with MK uptake (Figure 1B). Using Mini‐Mental State Examination (MMSE) scores as outcome, we observed that amyloid‐β negative individuals with high BMI showed worse cognitive performance as a function of both MK and FTP in the MTL, whereas individuals with vision impairment and hearing loss showed worse cognitive performance as a function of MK only (Figure 2A). Amyloid‐β positive individuals with hypercholesterolemia and hypertension presented worse cognitive performance as a function of both MK and FTP in the MTL (Figure 2B).

**Conclusion:**

In this preliminary analysis, sleep disorders, hypertension, and high BMI were independently associated with tau‐PET tracer uptake, with the effects varying according to amyloid‐β pathology. These prevalent factors in the elderly also changed the association between tau‐PET and cognition, underscoring the need for further studies to better understand their role in modulating this relationship.